# Potential distribution of pine wilt disease under future climate change scenarios

**DOI:** 10.1371/journal.pone.0182837

**Published:** 2017-08-10

**Authors:** Akiko Hirata, Katsunori Nakamura, Katsuhiro Nakao, Yuji Kominami, Nobuyuki Tanaka, Haruka Ohashi, Kohei Takenaka Takano, Wataru Takeuchi, Tetsuya Matsui

**Affiliations:** 1 Center for International Partnerships and Research on Climate Change, Forestry and Forest Products Research Institute, Matsunosato, Tsukuba, Ibaraki, Japan; 2 Tohoku Research Center, Forestry and Forest Products Research Institute, Nabeyashiki, Shimokuriyagawa, Morioka, Iwate, Japan; 3 Kansai Research Center, Forestry and Forest Products Research Institute, Nagaikyutaroh, Momoyama, Fushimi, Kyoto, Kyoto, Japan; 4 Faculty of International Agriculture and Food Studies, Tokyo University of Agriculture, Sakuragaoka, Setagaya, Japan; 5 Institute of Industrial Science, The University of Tokyo, Komaba, Meguro, Tokyo, Japan; Institute of Botany, CHINA

## Abstract

Pine wilt disease (PWD) constitutes a serious threat to pine forests. Since development depends on temperature and drought, there is a concern that future climate change could lead to the spread of PWD infections. We evaluated the risk of PWD in 21 susceptible *Pinus* species on a global scale. The MB index, which represents the sum of the difference between the mean monthly temperature and 15 when the mean monthly temperatures exceeds 15°C, was used to determine current and future regions vulnerable to PWD (MB ≥ 22). For future climate conditions, we compared the difference in PWD risks among four different representative concentration pathways (RCPs 2.6, 4.5, 6.0, and 8.5) and two time periods (2050s and 2070s). We also evaluated the impact of climate change on habitat suitability for each *Pinus* species using species distribution models. The findings were then integrated and the potential risk of PWD spread under climate change was discussed. Within the natural *Pinus* distribution area, southern parts of North America, Europe, and Asia were categorized as vulnerable regions (MB ≥ 22; 16% of the total *Pinus* distribution area). Representative provinces in which PWD has been reported at least once overlapped with the vulnerable regions. All RCP scenarios showed expansion of vulnerable regions in northern parts of Europe, Asia, and North America under future climate conditions. By the 2070s, under RCP 8.5, an estimated increase in the area of vulnerable regions to approximately 50% of the total *Pinus* distribution area was revealed. In addition, the habitat conditions of a large portion of the *Pinus* distribution areas in Europe and Asia were deemed unsuitable by the 2070s under RCP 8.5. Approximately 40% of these regions overlapped with regions deemed vulnerable to PWD, suggesting that *Pinus* forests in these areas are at risk of serious damage due to habitat shifts and spread of PWD.

## Introduction

Pine wilt disease (PWD) poses a serious threat to pine forests [1]. The pine wood nematode (PWN, *Bursaphelenchus xylophilus*) is the causal agent, while pine sawyer beetles (*Monochamus* spp.) act as a vector. PWN is thought to originate in North America [2], and thus, its wide distribution throughout the country is not associated with the epidemic disease [3], even though several *Pinus* species are susceptible to PWN [4]. In contrast, in Asia and Europe, PWN is an invasive pathogen introduced artificially from PWD-affected countries and subsequently causing damage to native *Pinus* trees [5,6,7].

PWD causes significant damage to forestry, local economies, and the ecologies of affected countries, degrading the quality and decreasing the quantity of pine wood products. In Japan, for example, the annual loss of pine wood as a result of PWD was more than 100 million m^3^ across 11 years between 1978 and 1988 and more than 50 million m^3^ between 1989 and 2014 [8]. The total financial loss, to date due to PWD in Japan is thought to be approximately 3700 million US dollars, assuming a market price of *P*. *densiflora* of US$ 100 / m^3^ [9]. From an ecological viewpoint, the loss of pine trees also reduces ecosystem functions and services, decreasing habitats for wild animals [10] and affecting soil erosion [5,11]. Once introduced into a region, PWN spreads rapidly to neighboring areas through vector beetles or accompanying human activity [11,12]. Control is therefore labor-intensive and costly [13], highlighting the importance of identifying vulnerable areas and prioritizing control measures.

The occurrence of PWD is associated with high temperatures and moisture deficits [5,14,15], the risk increasing with mean summer temperatures exceeding 20°C [4,15]. Modelling techniques aimed at evaluating PWD risk have been developed based on the dispersal capacity of the insect vector, human accidental transportation [7,16], and the physiological processes of host trees [17]. However, these studies focus on East Asia and Europe, with the potential PWD risk in other distribution areas of *Pinus* species remaining unclear.

The occurrence and spread of PWD is also likely to be affected by future climate change [18], which is thought to be highly probable according to the Intergovernmental Panel on Climate Change [19]. The increases in mean temperature and drought as a result of climate change could promote the spread of PWD into areas where the risk is low under current climatic conditions [18]. In addition, it has also been suggested that under cool climate conditions some PWNs, having infected *Pinus* trees, do not cause disease symptoms for a number of years [20,21]. Therefore, increasing temperatures in the future could also activate these latent nematodes. Understanding the changing risks with climate change will therefore facilitate the establishment of measures aimed at prevention of future PWD outbreaks [18]. However, few studies have yet to evaluate the resulting risks under future climate change scenarios.

Climate change will also have direct effects on the growth of *Pinus* forests, with increasing temperatures negatively affecting the forest growth via aridification of regions where droughts is the primary constraint of growth and productivity [22]. Thus, changes in temperature and precipitation constitute a significant threat to *Pinus* forests, even if undamaged by PWD. Increasing temperatures also affect host susceptibility to pathogens [23,24]. Thus, areas where habitat suitability for *Pinus* species is likely to decrease in the future will be at high risk of PWD occurrence. Projecting the impact of climate change on both PWD risk and habitat suitability in *Pinus* distribution areas is therefore important in terms of understanding those areas vulnerable to PWD.

The aims of the present study were to evaluate the PWD risk in 21 *Pinus* species on a global scale and determine current and future changes in vulnerable regions with climate change. The MB index [25], which represents the sum of the difference between the mean monthly temperature and 15 when the mean monthly temperature exceeds 15°C, was used to determine vulnerable regions under current and future climate conditions. We also evaluated the impact of climate change on habitat suitability for each of the 21 *Pinus* species then integrated all the findings in order to discuss the potential risk of PWD spread under future climate change.

## Methods

### The global distribution of PWD

Globally, Asia is most severely affected by PWD. In Japan, the mass mortality of *P*. *thunbergii* and *P*. *densiflora* that occurred in Nagasaki City, Kyushu Island, during the 1900s [26] was considered the first reported case of PWD, and despite intensive efforts to control this epidemic disease, PWD spread from western to northeastern Japan throughout the 20^th^ century [27]. PWD is now observed on three of the main islands of Japan, excluding the northern island of Hokkaido. Epidemic PWD has also occurred in South Korea, mainland China, and Taiwan since the 1970s or 80s [28–30]. In South Korea, despite strenuous efforts aimed at control, PWD has spread nationwide from southern *P*. *densiflora* and *P*. *thunbergii* forests to northern *P*. *koraiensis* forests [28]. In mainland China, PWD was first identified in a *P*. *thunbergi*i forest in Nanjing City thereafter spreading to *P*. *massoniana* forests widely distributed throughout the country, and currently prevalent in most of the south-western provinces [29]. In Taiwan, serious PWD outbreaks have also occurred since 1985 [30], recently, spreading to 14 provinces and cities, causing death of more than 0.5 billion pine trees [31]. In Europe, native *Pinus* species that are widely cultivated such as *P*. *sylvestris* and *P*. *pinaster* are known to be susceptible to PWD [32,33], and therefore the European and Mediterranean Plant Protection Organization (EPPO) recommends the prohibition of imported softwood products unless they properly treated to exterminate PWN [6,34]. Nevertheless, despite these restrictions, outbreaks of PWD occurred in Portugal in1999 and more recently in Spain [12,35–37].

### Target species

**Fig 1:**
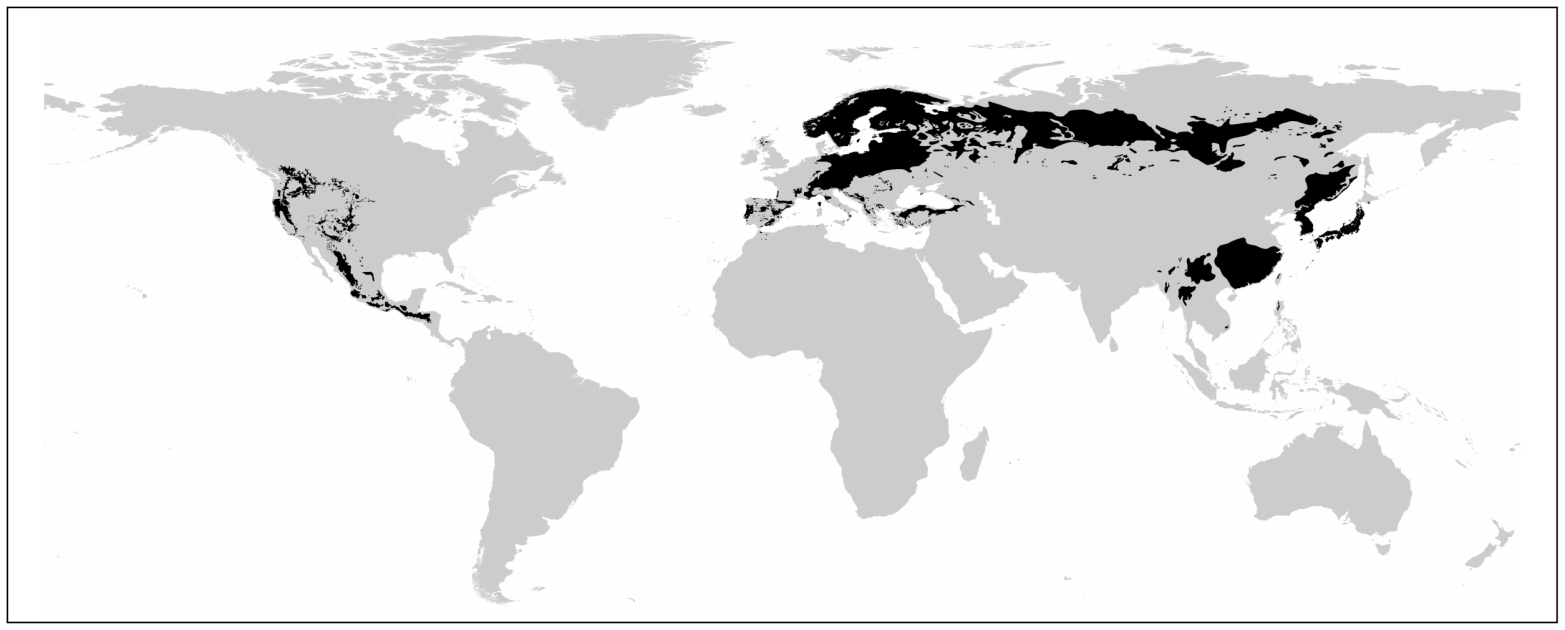
Distribution map of the 21 susceptible *Pinus* species [40].

Different *Pinus* species differ in their susceptibility to PWN [5,14,32,33,38]. In this study, we targeted 21 species (Table 1) evaluated as “susceptible” by Mamiya [14] or Evans [5], or “susceptible” to “highly susceptible” by Nakamura and Tabata [39]. Next, we evaluated the PWD risk within their native distribution areas as proposed by Critchfield and Little [40]. The set of distribution information was manually digitized and stored in geographical information systems (GISs; Fig 1).

**Table 1:** PWN-susceptible *Pinus* species.

Asia	Europe	North America
*P*. *koraiensis*	*P*. *sylvestris*	*P*. *ponderosa*
*P*. *densiflora*	*P*. *mugo*	*P*. *strobiformis*
*P*. *parviflora*	*P*. *pinaster*	*P*. *muricata*
*P*. *thunbergii*	*P*. *nigra*	*P*. *radiata*
*P*. *luchuensis*		*P*. *leiophylla*
*P*. *massoniana*		*P*. *engelmannii*
*P*. *yunnanensis*		*P*. *oocurpa*
*P*. *insularis*		*P*. *hartwegii*
		*P*. *ayacahuite*

### Calculation of the MB index

In Japan, the MB index was proposed to explain the known distribution of PWD-affected areas [25]. According to the concept of effective accumulative temperature, the MB index represents the sum of the difference between the mean monthly temperature (Tm) and 15 when the mean monthly temperature exceeds 15°C; that is, ∑(Tm-15) when Tm ≥ 15. Originally, it was thought that severe PWD damage would not occur when the MB index is below 40°C months; however, this hypothesis was proven invalid by subsequent prevalence in such areas. The relationship between the level of damage due to PWD and MB index in several areas where PWD has fully spread was therefore re-examined, and a new threshold of 22°C months for the occurrence of severe PWD damage in *P*. *densiflora* forests was set [41]. As a result, this new MB threshold has been used to successfully explain the range limit of PWD in the affected areas in Japan. Owing to the ease of calculation and the predictive accuracy, the MB index is now regarded as a powerful tool for evaluation of PWD risk, although the differences in susceptibility among *Pinus* species have yet to be considered.

A dataset of mean monthly temperatures under current and future climate conditions was prepared using the WorldClim dataset (http://www.worldclim.org). For the current MB index, the 30-year mean monthly temperatures between 1960 and 1990 were used, while for future MB indices, a set of 20-year mean monthly temperatures between 2041 and 2060 (hereafter, the 2050s) and 2061 and 2080 (hereafter, the 2070s) was determined by averaging the minimum and maximum temperatures provided by WorldClim. The spatial resolution of the present study was set at 30″ (approximately 1 km^2^ spatial resolution at the equator).

In the present study, we attempted to compare the differences among four different representative concentration pathways (RCPs) and two future time periods. RCPs represent a set of greenhouse gas concentrations and emission pathways [42], a higher RCP representing larger emissions and more rapid global warming. We subsequently calculated eight MB indices for the four different RCPs (2.6, 4.5, 6.0, and 8.5) and two time periods (2050s and 2070s). For the future MB indices, it is important to consider the uncertainty among the general circulation models (GCMs). We therefore used five GCMs of the World Climate Research Program’s (WCRP’s) Coupled Model Intercomparison Project Phase 5 (CMIP5) multi-model dataset, which is available in the WorldClim dataset. The five GCMs were as follows: GFDL-CM3, HadGEM2-ES, IPSL-CM5A-LR, MIROC-ESM-CHEM, and NorESM1-M. Finally, MB indices calculated using the five GCMs were averaged for each RCP and each time period, thereby resulting in eight future MB indices (four for the 2050s and four for the 2070s) prepared for the following analysis.

### Evaluation of regions vulnerable to PWD

According to the isograms of the MB index, areas falling within the natural distributions of susceptible *Pinus* species were classified into the following three risk categories: 1) regions of low vulnerability where the MB index is < 19°C months, 2) moderately vulnerable regions where the MB index is ≥ 19°C and < 22°C months, and 3) vulnerable regions where the MB index is ≥ 22°C months [41]. The MB index for classifying areas at risk was originally prepared for *P*. *densiflora* forests, with reference to decreasing levels of damage where PWD had fully spread after a long history of infestation. However, this situation is not expected for other *Pinus* species, and therefore, setting the threshold indices for each species poses a challenge. We therefore extrapolated the thresholds for *P*. *densiflora* to other species to evaluate PWD risks on a global scale.

Vulnerable regions under current and future climate conditions were subsequently mapped for the four RCPs (2.6, 4.5, 6.0, and 8.5) and the area of vulnerable regions was calculated for all and respective *Pinus* species. In calculating the area of vulnerable regions, we masked regions by forest land since the distribution areas of some *Pinus* species were drawn as large polygons containing many non-forest areas. The forest land mask was created using the 2001 Moderate Resolution Imaging Spectroradiometer (MODIS) land cover map classified by the International Geosphere Biosphere Programme (IGBP) land cover classification derived from the Global Land Cover Characteristics Database Version 2.0. Areas falling within the forest categories were used as the forest land mask.

### Changes in habitat suitability for *Pinus* species

**Fig 2:**
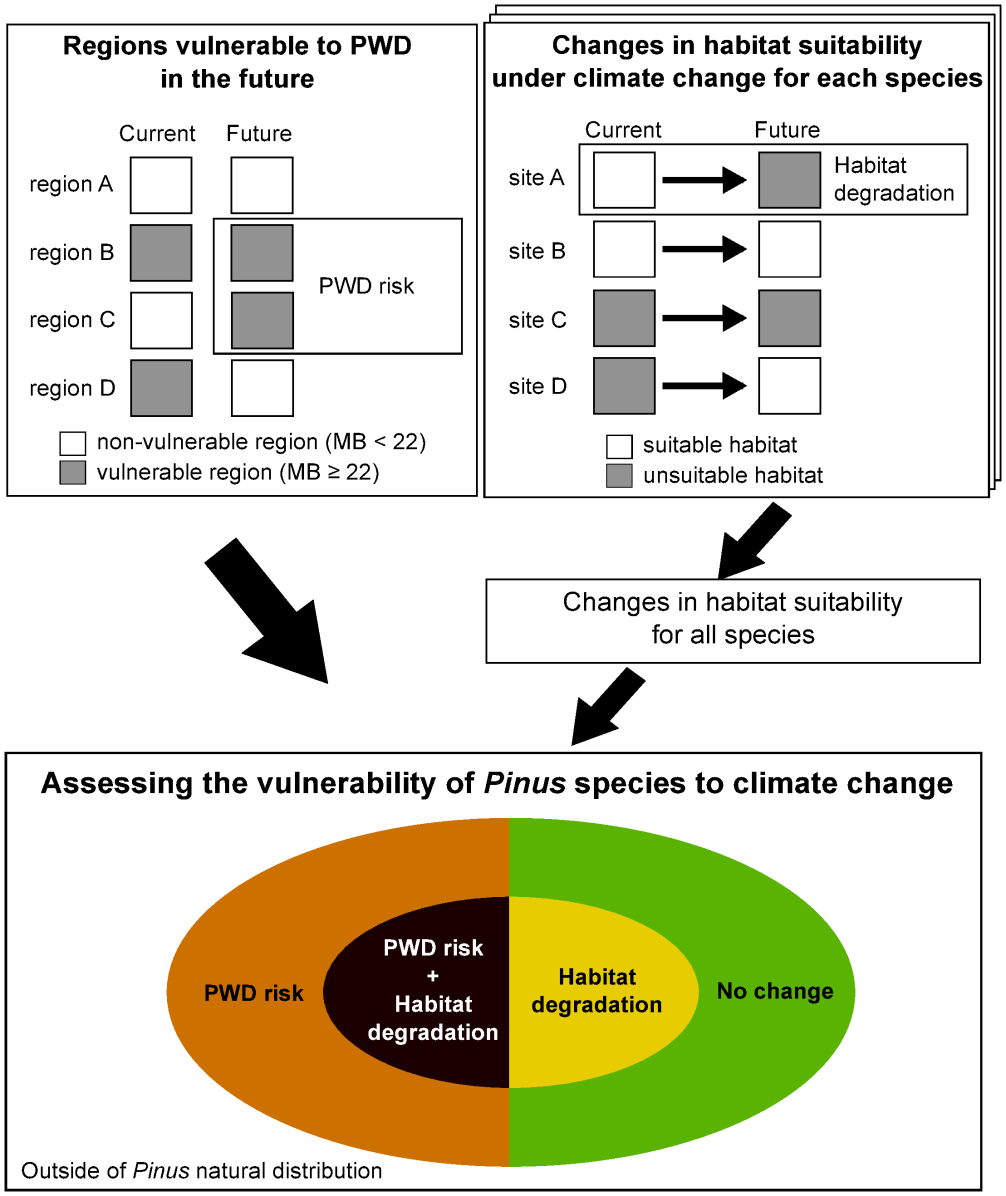
Conceptual scheme of the present study.

The effects of climate change on habitat suitability for *Pinus* species were evaluated using species distribution models aimed at estimating the relationship between species occurrence at a site and the environmental and / or spatial characteristics of this site [43]. We estimated the potential distribution areas of *Pinus* species under current and future (2070s) climatic conditions then evaluated the changes in habitat suitability with climate change in their natural distribution areas. Areas in which conditions may become climatically unsuitable in the future despite being currently suitable were evaluated as vulnerable (Fig 2). Because the migration of *Pinus* species up till the 2070s was not thought to be considerable, we evaluated future changes in habitat suitability in their natural distribution areas only.

The effects of climate change on habitat suitability were evaluated for 18 susceptible *Pinus* species, excluding *P*. *radiata*, *P*. *luchuensis*, and *P*. *muricata*, whose distributions are localized (coastal and island areas). Occurrence data for these species were derived from Critchfield and Little [40], who projected species distributions as points and areas. Point data were changed to digital data using GIS, and area data to digital point data as described below. First, we changed area data into grid data, which were masked by forest area. Random points were then generated within these grid data (1 point / 2,000 km^2^). A total of 100 points were generated, even if the grid area of a given species was ≤ 2,000 km^2^.

The 19 bioclimatic variables downloaded from the WorldClim dataset with 30″ resolution (www.worldclim.org) [44] were used to determine current and future climate data. Future climate scenarios were the same as those used in the MB index calculations (five GCMs and four RCPs). In addition to these bioclimatic variables, we used an aridity index as an environmental variable, because growth and mortality of *Pinus* trees is greatly affected by drought conditions [45]. The aridity index was calculated as the ratio of mean annual precipitation (P) and mean annual potential evapotranspiration (PET) as follows [46]:

$$\text{AI}=\;\frac{P}{PET}$$

using the mountain microclimate simulation model (MTCLIM) [47], which has been used to estimate and extrapolate daily temperature, precipitation, humidity, and radiation over large regions of complex terrain [48]. The acceptable performance of this scheme has also been assessed [49,50]. We included only variables with a Pearson correlation coefficient ≤ 0.70 and with high ecological importance in the predictive model; namely, mean diurnal range, maximum temperature of the warmest month, minimum temperature of the coldest month, mean temperature of the wettest quarter, precipitation seasonality, precipitation of the warmest quarter, precipitation of the coldest quarter, and the aridity index.

Distribution models were generated using the maximum entropy model (Maxent ver 3.3) [51], which uses presence-only data to predict the distribution of a species based on the theory of the maximum entropy method [51]. The performance of the Maxent model is influenced by certain parameters [51]. In the present study, we therefore changed some parameters from the default settings. In the feature setting, which consists of environmental variables and functions thereof [51], all features were used in each model. The regularization parameter (betamultiplier) was set to 2.5 to facilitate the creation of less complex and smoother models and to avoid over-fitting of parameters to the evaluation dataset [52].

A total of 20,000 random background points were generated in a bias grid showing the variations in grid area as sampling weights [52]. These background points were restricted to terrestrial ecoregions categorized as eight bio-geographic realms and 14 biomes [53] in which species occurrence was contained. This was to exclude areas containing no remaining habitat [54,55].

Five-fold cross-validation was used for the assessment of model performance, and averaged AUC [area under the receiver operating characteristic (ROC) curve] across the five iterations was used to evaluate model performances for each species. Occurrence probability was converted into a binary (suitable versus unsuitable habitat) using a threshold, thereby maximizing the sum of sensitivity and specificity, which has been proven valid for use with presence-only data such as Maxent [56]. For future models, we constructed models for five GCMs under each RCP, and averaged the occurrence probabilities of the five models. Thereafter, we detected potential vulnerable habitats wherein habitat suitability changed from suitable in the current to unsuitable in the future (Fig 2).

Finally, we integrated the results of both the PWD risk and habitat degradation and evaluated the vulnerability of *Pinus* species to climate change. A conceptual scheme of our method of analysis is depicted in Fig 2.

## Results

### Regions currently vulnerable to PWD

**Fig 3:**
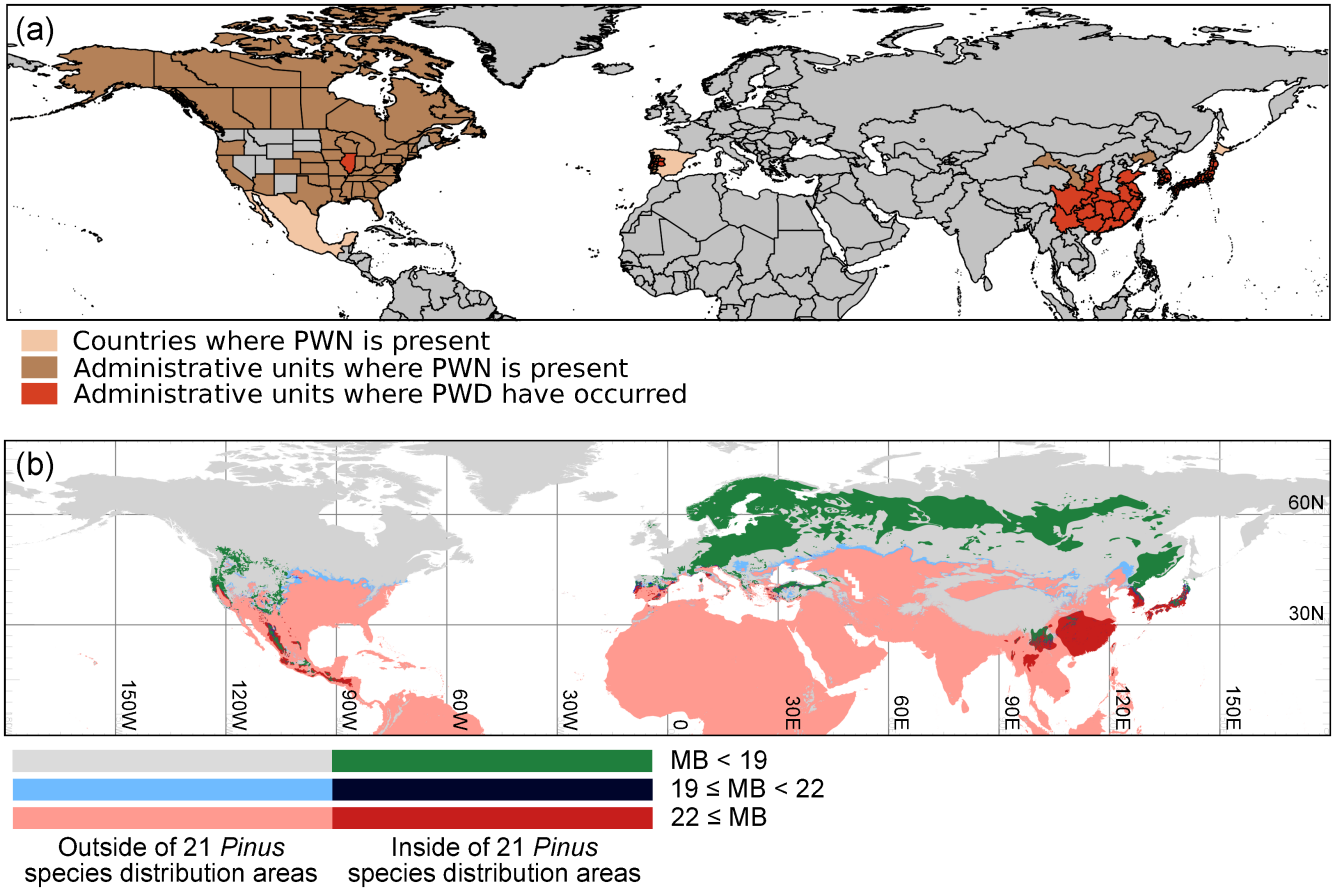
Distribution map of PWN and PWD (a) and regions vulnerable to PWD under current climate conditions (b). Distributions of PWN were drawn according to EPPO [57]. For Mexico, Spain, Taiwan, Korea, and Japan, the presence of PWN was recorded at the country level [57]. Occurrences of PWD were determined according to previous literature [29,30,35–39,58,59].

**Fig 4:**
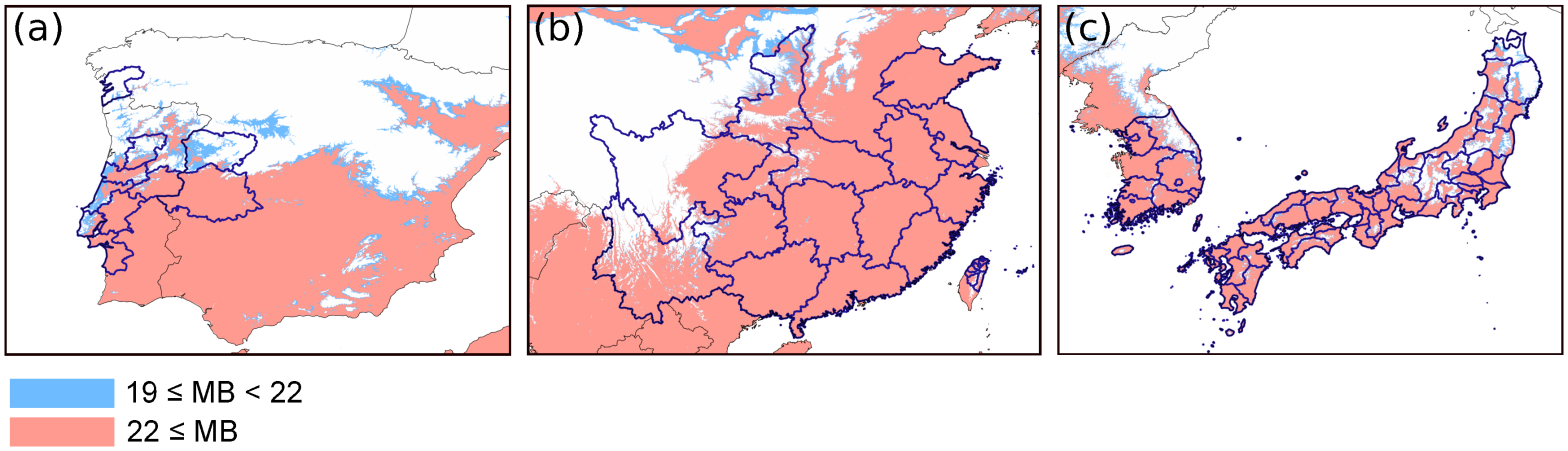
Details of risk areas in Europe (a), mainland China and Taiwan (b), and South Korea and Japan (c). Blue lines indicate the provinces where PWD has been reported.

Fig 3 and S1 Fig show the current distributions of PWN [57] and PWD [29,30,35–39,58,59] and the regions currently vulnerable to PWD. Within the natural *Pinus* distribution area, the southern parts of North America, Europe, and Asia were categorized as vulnerable regions (MB ≥ 22), while in Asia, large parts of the *Pinus* distribution area were categorized as vulnerable. Representative provinces in which PWD has been reported at least once [29,30,35–39,58,59] overlapped with the regions categorized as vulnerable to PWD (Fig 4).

S2 Fig represents the area ratio of vulnerable regions to natural distribution areas of *Pinus* species masked by forest areas for each country. Under current climate conditions, the area ratio of vulnerable regions was high in East Asia, while in the future, the ratio increased in several countries in Europe, North Asia, and North America.

### Regions of future vulnerability to PWD

**Fig 5:**
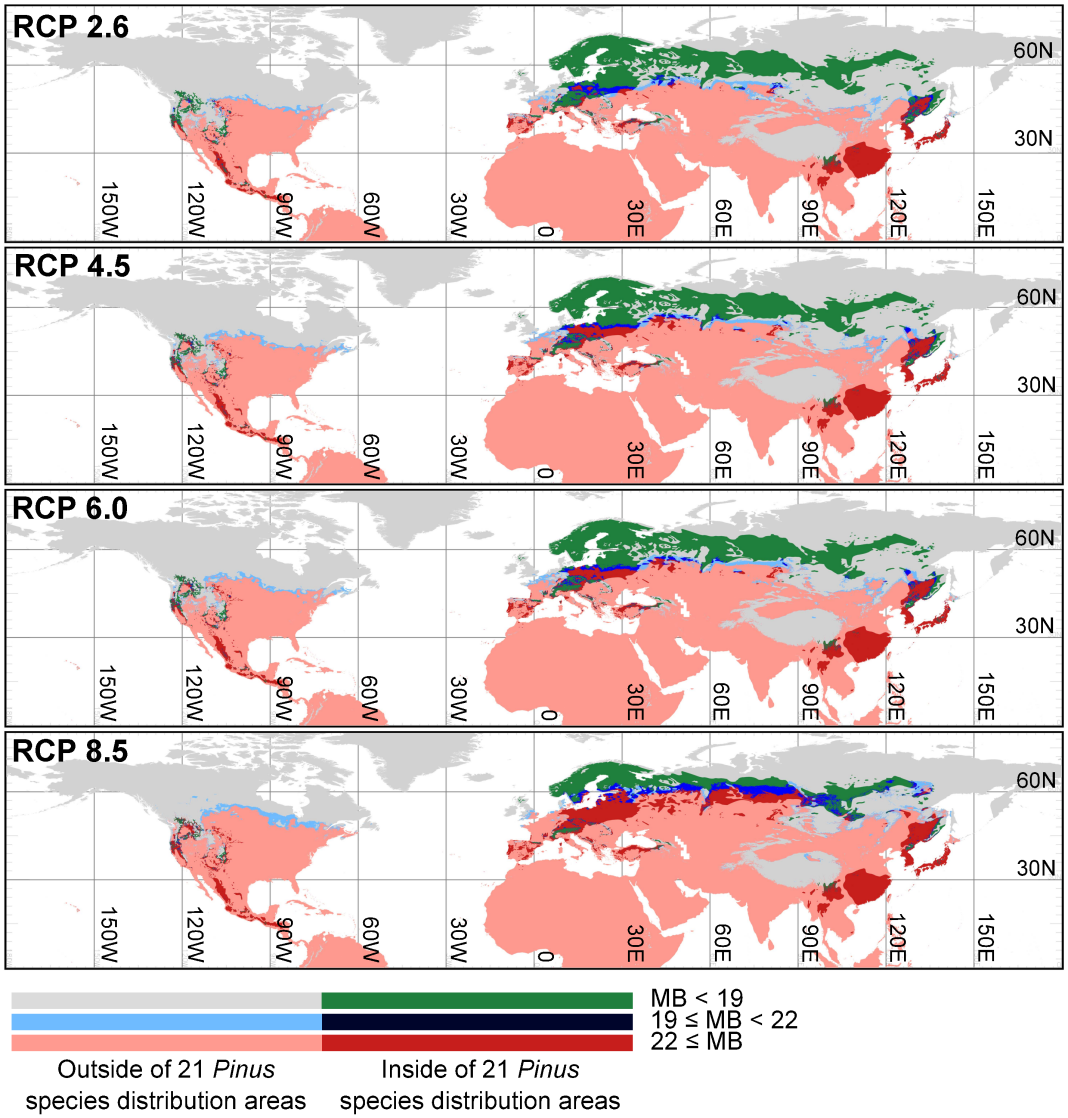
Regions vulnerable to PWD under future climate conditions according to four RCPs (2.6, 4.5, 6.0, and 8.5) in the 2070s.

Under each RCP scenario, vulnerable regions expanded toward northern parts of Europe, Asia, and North America up till the 2070s (Fig 5 and S3 Fig). *Pinus* distribution areas in Portugal and Spain, two countries in Europe affected, were categorized as vulnerable under each of the four RCP scenarios. Under RCP 8.5, vulnerable regions expanded considerably in northern Asia compared with RCPs 2.6, 4.5, and 6.0. In East Asia, the spatial extent of vulnerable regions did not differ considerably among the four RCPs, except in far-east Russia. In North America, even the northern region of the *Pinus* distribution areas was categorized as vulnerable under RCP 8.5.

### Changes in areas of vulnerable regions with climate change

**Fig 6:**
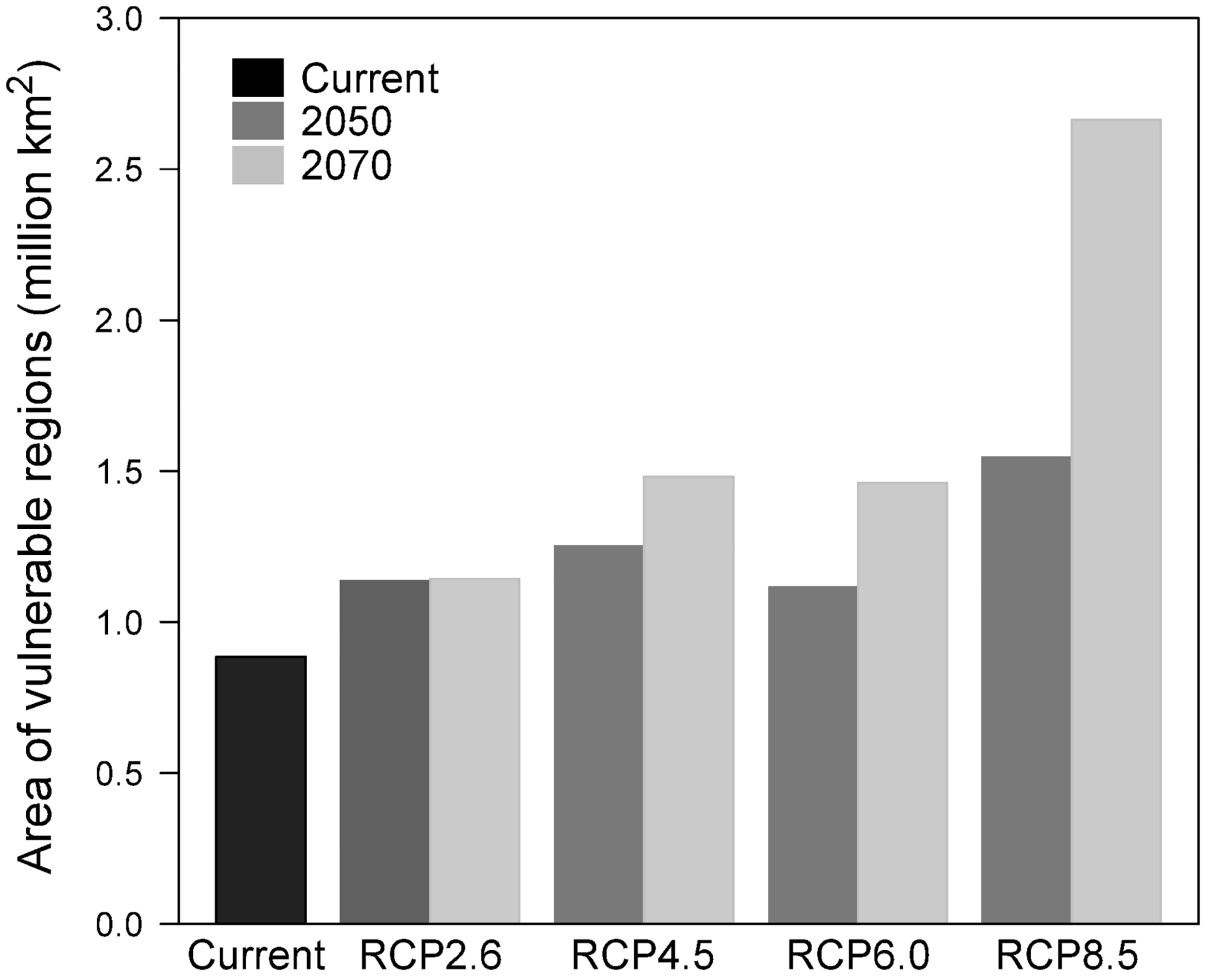
Changes in the areas of vulnerable regions with climate change under four RCPs (2.6, 4.5, 6.0, and 8.5) in the 2050s and 2070s.

The total area of vulnerable regions (MB ≥ 22) under current climatic conditions within *Pinus* distribution areas was 0.88 million km^2^, approximately 16% of the total *Pinus* distribution area (5.38 million km^2^; Fig 6). Under future climate conditions, areas of vulnerable regions increased with increasing RCP. Under RCP 2.6, the areas of vulnerable regions increased little from the 2050s to 2070s (1.13 and 1.14 million km^2^, respectively); however, under RCP 8.5, a considerable increase from 1.54 to 2.66 million km^2^ was observed.

### Differences in PWD risk among susceptible *Pinus* species

**Fig 7:**
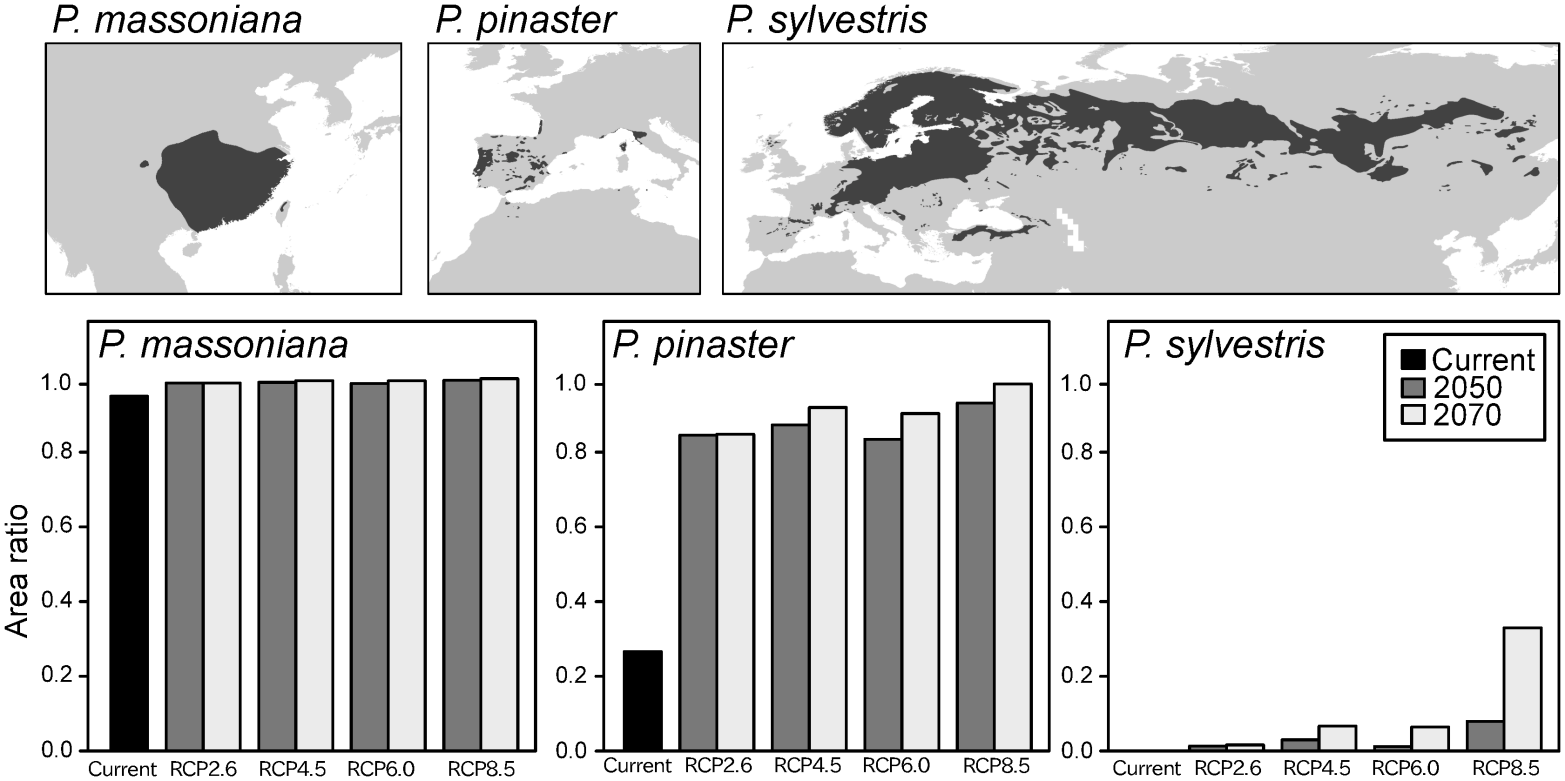
Example changes in the areas of vulnerable regions of three *Pinus* species (*P*. *massoniana*, *P*. *pinaster*, and *P*. *sylvestris*). Upper figures represent native distribution areas of each species and lower figures the area ratio of vulnerable regions to the whole distribution area under each climate condition for each species.

Differences in the PWD risk exist among the target *Pinus* species, and the magnitude of increase in vulnerable regions under each RCP scenario differed considerably among the 21 target species. Using 50% of the native distribution area as the threshold area ratio of vulnerable regions, we categorized each of the 21 *Pinus* species into three groups as follows (Table 2 and Fig 7):

1. High-risk species: Species for which ≥ 50% of their natural distribution area was categorized as vulnerable under both the current climate conditions and any one of the four future RCP scenarios.
2. Future-risk species: Species for which ≥ 50% of their natural distribution area was categorized as non-vulnerable under current climate conditions, but categorized as vulnerable under any one of the four future RCP scenarios.
3. Low-risk species: Species for which ≥ 50% of their natural distribution area was categorized as non-vulnerable under both the current climate conditions and all four future RCP scenarios.

**Table 2:** PWD risk of each susceptible *Pinus* species.

	High risk	Future risk	Low risk
Asia	*P*. *densiflora*	*P*. *koraiensis*	
	*P*. *thunbergii*	*P*. *parviflora*	
	*P*. *luchuensis*	*P*. *yunnanensis*	
	*P*. *massoniana*		
	*P*. *insularis*		
Europe		*P*. *pinaster*	*P*. *sylvestris*
		*P*. *nigra*	*P*. *mugo*
North America	*P*. *engelmannii*	*P*. *strobiformis*	*P*. *ponderosa*
	*P*. *oocurpa*	*P*. *radiata*	*P*. *muricata*
	*P*. *ayacahuite*	*P*. *leiophylla*	
		*P*. *hartwegii*	

As a result, five Asian *Pinus* species including *P*. *massoniana*, *P*. *thunbergii*, and *P*. *densiflora* were categorized as high-risk species (Table 2), with no low-risk species identified in Asia. In contrast, two low-risk species (*P*. *mugo* and *P*. *sylvestris*) and no high-risk species were identified in Europe. In North America, three high-risk species, four future-risk species, and two low-risk species were identified (Table 2).

### Changes in habitat suitability

**Fig 8:**
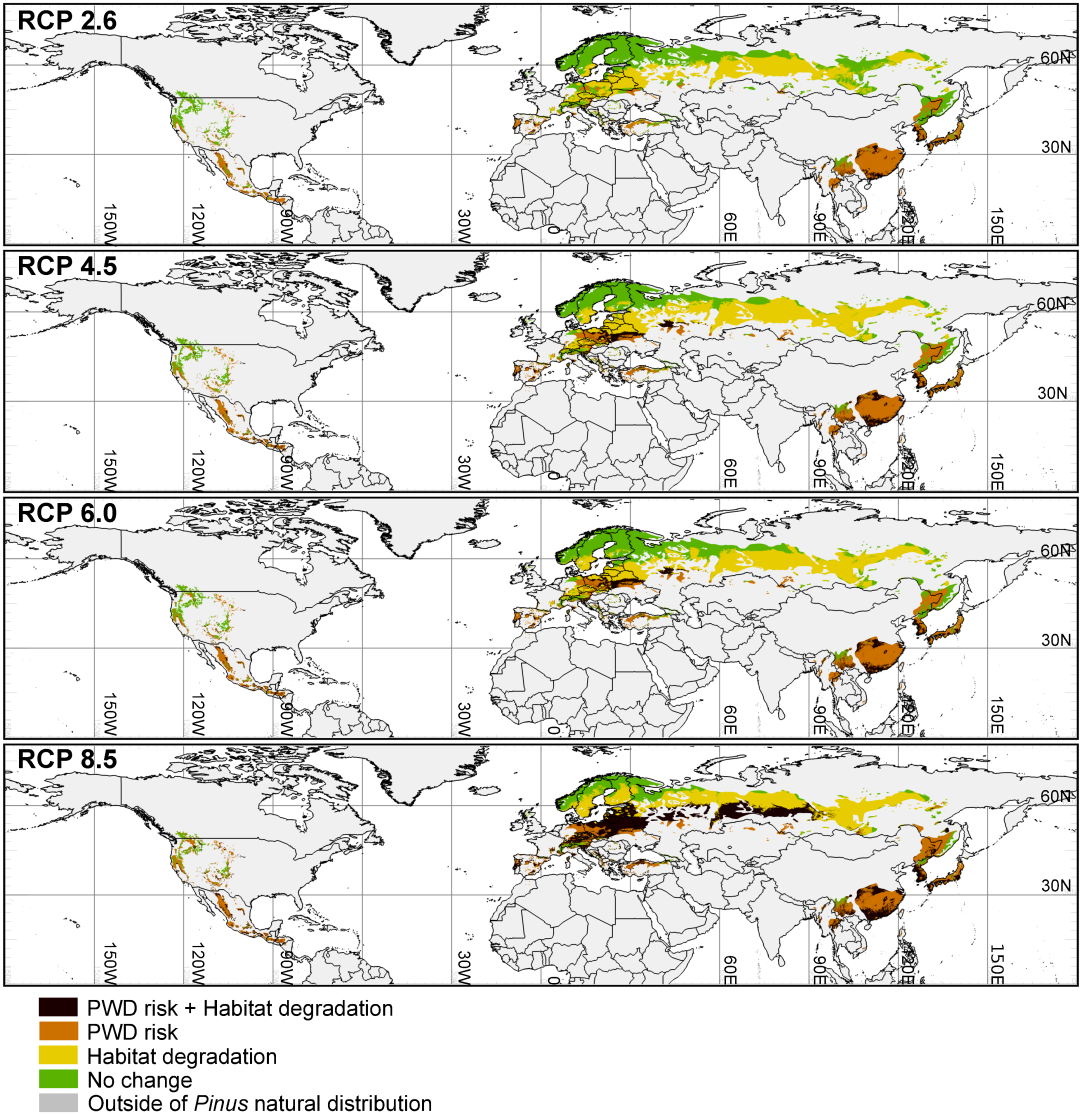
Regions vulnerable to PWD and habitat degradation for susceptible *Pinus* species under climate change in the 2070s. Areas integrating dark brown and yellow indicate regions vulnerable to habitat degradation.

The AUC values of Maxent models for each *Pinus* species ranged between 0.882 and 0.999. Fig 8 and S4 Fig show the potential vulnerable habitats of 18 *Pinus* species under future climate change. Under RCP 2.6, the climate conditions in some *Pinus* distribution areas in Europe and Asia will become unsuitable in the 2070s for susceptible *Pinus* species (dark brown and yellow in Fig 8). Most of these vulnerable habitats did not overlap with the regions vulnerable to PWD (yellow in Fig 8). A higher RCP scenario resulted in an increase in vulnerable habitats, while under RCP 8.5, large parts of the *Pinus* distribution areas in Europe and Asia will become unsuitable, with approximately 40% of these areas overlapping with vulnerable regions (dark brown in Fig 8).

## Discussion

### Regions currently vulnerable to PWD

Regions identified as vulnerable to PWD using the MB index largely overlapped with historical and current PWD-affected regions, although the range of vulnerable regions in Europe was slightly small compared with previous studies [17,18]. This suggests the applicability of the MB index in evaluating PWD risks for non-*P*. *densiflora* forests on a global scale.

In particular, large areas of vulnerable regions were detected in Asia, where a large percentage of the *Pinus* forest has already been damaged by PWD. Comparisons of the areas of vulnerable regions among susceptible *Pinus* species also indicated that several high-risk species are Asian *Pinus* species (Table 2). Management measures aimed at preventing PWN infections have been implemented in Japan, South Korea, and mainland China such as felling and fumigation of dead trees in infested forests, strengthening of quarantine measures, and spraying or trunk injection of chemical agents [28,29]. These measures have reduced PWD spread and damage in some regions [13,28].

In Europe, only southern regions were categorized as vulnerable under current climate conditions. In contrast, in northern European forests, the epidemic risk of PWN is relatively low, even if PWN were to be introduced into these areas. These results are consistent with previous studies [4,18]. Measures aimed at minimizing further introduction of PWD are therefore important in southern Europe.

In North America, the distribution of PWN cannot be equated with that of PWD [3]; however, some exotic susceptible *Pinus* species such as *P*. *sylvestris*, *P*. *nigra*, and *P*. *ponderosa* have been planted in North America [60]. If these forests are located in currently vulnerable regions, urgent interventions aimed at controlling PWD would therefore be necessary.

### Changes in vulnerable regions under climate change

Regions vulnerable to PWD were projected to expand towards northern parts of Europe, Asia, and North America under each RCP climate change scenario; however, the range of expansion differed (Fig 5 and S3 Fig). This indicates that the risk of PWD outbreaks in new areas depends on the extent of mitigation of greenhouse gas emissions. Even if greenhouse gas emissions are reduced to limit the increase in global average temperatures to < 2°C above pre-industrial levels (i.e., RCP 2.6), vulnerable regions will spread further north. However, it should be noted that because the expansion of PWD is affected not only by climate factors, but also by the dispersal capacity of insect vectors, the transportation of infected logs to non-infected regions, and susceptibility of host trees [7,16,17], the vulnerable regions projected in our study do not necessarily represent realistic expansion of PWD throughout these regions. Nevertheless, the risk of PWD outbreaks in these regions will increase with global climate change.

Under RCP 2.6, in particular, wide-spread expansion of vulnerable regions was projected in northeastern parts of Asia. *P*. *koraiensis* is distributed in this area, where it forms the principal component of old-growth forests [61]. Furthermore, *P*. *koraiensis* is also one of the major plantation tree species in South Korea, Russia, and mainland China, and is highly valued for its wood products and nuts [62,63]. Damage to these forests by PWD would result in a largely modified forest structure, and consequently, forestry industries in these regions would be seriously damaged. On the other hand, in Portugal and Spain, two PWD-affected countries in Europe, almost all *Pinus* distribution areas were categorized as vulnerable to PWD in the future. Increased attention to future PWD risks in these regions is therefore required, even though establishment of PWN has been limited under current climatic conditions.

Under the highest RCP scenario in which CO_2_ concentrations will continue to increase till the year 2200 (RCP 8.5), regions vulnerable to PWD are likely to become wide-spread across Europe and Asia, resulting in serious economic and ecological damage. In northern Europe, the forestry industry is primarily based on native tree species, including *Pinus* species such as *P*. *nigra*, *P*. *pinaster*, and *P*. *sylvestris* [64]. Our study suggests that the majority of distribution areas of *P*. *nigra* and *P*. *pinaster* will become vulnerable to PWD in the future, despite the low risk under current climate conditions. PWD damage of these species would seriously affect the European forestry industry. Moreover, in northwestern Russia, rare old-growth natural forests have been preserved [64], and *P*. *sylvestris* is the primary species of such forests [4,64]. If such forests were infected by PWD, the ecosystem structure of these valuable old-growth forests would be seriously modified. Boreal forests are also primary carbon sinks [65], particularly *P*. *sylvestris* forests in Siberia and European Russia [65]. The spread of PWD in these areas would therefore change these forests into carbon sources with the death of large numbers of *Pinus* trees.

### Impact of climate change on habitat suitability for *Pinus* species

The current study also determined the negative effect of climate change on habitat suitability in major *Pinus* distribution areas, particularly in Eastern Europe and Asia (dark brown and yellow in Fig 8). Climate change is predicted to have a negative impact on forest growth and products in Southern and Eastern Europe via increased drought and disturbance risks, despite some positive effects in Northern and Western Europe [66]. Furthermore, in dry European forests where *P*. *sylvestris* dominates, climate change is expected to accelerate successional replacement of *P*. *sylvestris* with slow-growing tree species such as *Picea abies* [67]. Our results are consistent with the results of these previous studies.

Under RCP 2.6, almost all boreal *Pinus* forests fell outside the regions vulnerable to PWD in the 2070s. However, estimates of the effect of climate change revealed a decrease in habitat suitability in some areas, probably due to increasing temperatures and aridification. Climate change may therefore result in a decline in these boreal *Pinus* trees, despite the low risk of PWD damage. Moreover, under RCP 8.5, vulnerable habitats for *Pinus* species expanded not only in Europe and North Asia but also in East Asia, approximately 40% of these areas overlapped with the regions deemed vulnerable to PWD. These findings suggest that these regions are at higher risk than other vulnerable regions containing non-vulnerable habitats.

### Adaptation to PWD risk with global warming

Adaptive management is required to limit the spread of PWD and conserve ecosystem functions of *Pinus* forests with climate change. This is necessary not least because even if greenhouse gas emissions are reduced to the level assumed under RCP 2.6, global warming will continue to progress. We categorized natural distribution areas of susceptible *Pinus* species with climate change into four vulnerability types by integrating the PWD risk and predicted habitat degradation in each region (Fig 8). The following are adaptation measures for each vulnerability type:

1. Regions vulnerable to PWD and habitat degradation
  Our results suggest that regions of this type, in which the risk of a PWD outbreak is particularly high, are widely distributed in East Europe and Asia under RCP 8.5 (dark brown in Fig 8). In East Europe and North Asia, most of these regions are not adjacent to current PWD-affected regions, and thus, preventing the artificial introduction of PWN is one of the most important adaptation measures. The introduction of PWN into non-native areas is primarily associated with trade and global flow of forest products [7,68,69]; thus, it is important to understand the possible pathways of PWN and prevent the import of infected forest products. In *Pinus* plantation forests, logging of pine trees prior to the occurrence of PWD and reforestation of non-*Pinus* species might be valuable in avoiding unexpected PWD spread and conserving forest ecosystems, not least because environmental conditions in these regions may become unsuitable for *Pinus* species due to climate change.
2. Regions vulnerable to PWD
  It was predicted that regions of this type are primarily distributed in South Europe and East Asia, overlapping or near to current PWD-infected regions (orange in Fig 8). Thus, adaptation measures aimed at preventing further spread of PWN, such as logging and fumigation or burning of infected trees [28,29], are recommended. If, however, PWD spread does occur, programs aimed at reforestation of damaged forests may be necessary. Unlike the regions vulnerable to both PWD and habitat degradation, these regions would not suffer the effects of habitat degradation due to climate change. Thus, in regions where *Pinus* species play an important role in forestry and culture, reforestation of *Pinus* forests might be possible if the total eradication of PWN from the site is successful prior to reforestation. Reforestation using resistant pines, which have been bred in Japan and mainland China [70,71], may also be effective.
3. Regions vulnerable to habitat degradation
  In regions of this type (yellow in Fig 8), the PWD risk in the future is relatively low; however, our results predict that climate change will result in deterioration of habitat conditions for *Pinus* species. Although responses of each *Pinus* species to climate change remain uncertain, it has been suggested that drought and warming decrease growth and increase mortality of *Pinus* trees [45]. Continuous monitoring of the effects of climate change on *Pinus* populations is therefore recommended, even though it is difficult to distinguish the effects of climate alone and the results of natural succession. Climate change might induce a range shift in some *Pinus* species outside of their current distribution areas. In this study, we evaluated changes in habitat suitability within natural distribution areas only. Protection of migration routes surrounding current distribution areas might therefore be a valuable adaptation strategy.
4. No-change regions
  *Pinus* forests of this type (green in Fig 8) will not be affected by the spread of PWD or habitat degradation caused by climate change. These regions are therefore likely to remain stable during climate change and will become key regions for the conservation of *Pinus* forests. In particular, in valuable old-growth *Pinus* forests, management plans aimed at avoiding artificial alteration such as the establishment of protected areas is recommended.

Overall, our study suggests that future climate change scenarios are likely to result in serious PWD-related damage and habitat degradation to global *Pinus* forests. To develop suitable management strategies, it is therefore important to understand the functional roles of *Pinus* forests and reach a consensus on the functions these *Pinus* forests should perform. Repeated and consistent monitoring of the impacts of climate change on *Pinus* forests are therefore required, not only from an economic viewpoint, but also in terms of ecosystem structure and services.

## Supporting information

S1 Fig Distribution maps of PWN and PWD in Europe (a) and Asia (b), and the regions vulnerable to PWD under current climate conditions in Europe(c) and Asia (d). Distributions of PWN were determined according to EPPO [56], and occurrences of PWD in accordance to previous literature [29,30,34–38,57,58].
(TIF)

S2 Fig Area ratios of vulnerable regions to natural distribution areas of *Pinus* species masked by forest areas. (TIF)

S3 Fig Regions vulnerable to PWD under future climate conditions according to four RCPs (2.6, 4.5, 6.0, and 8.5) in the 2070s in Europe (left side) and Asia (right side). (TIF)

S4 Fig Regions vulnerable to PWD and habitat degradation for susceptible *Pinus* species under climate change in the 2070s in Europe (left side) and Asia (right side). Areas integrating dark brown and yellow indicate regions vulnerable to habitat degradation.
(TIF)
