# Supplementary figures and images for: Potential distribution of pine wilt disease under future climate change scenarios
Source: PLoS One. 2017 Aug 10;12(8):e0182837. doi: 10.1371/journal.pone.0182837 (PMC5552256; doi:10.1371/journal.pone.0182837)

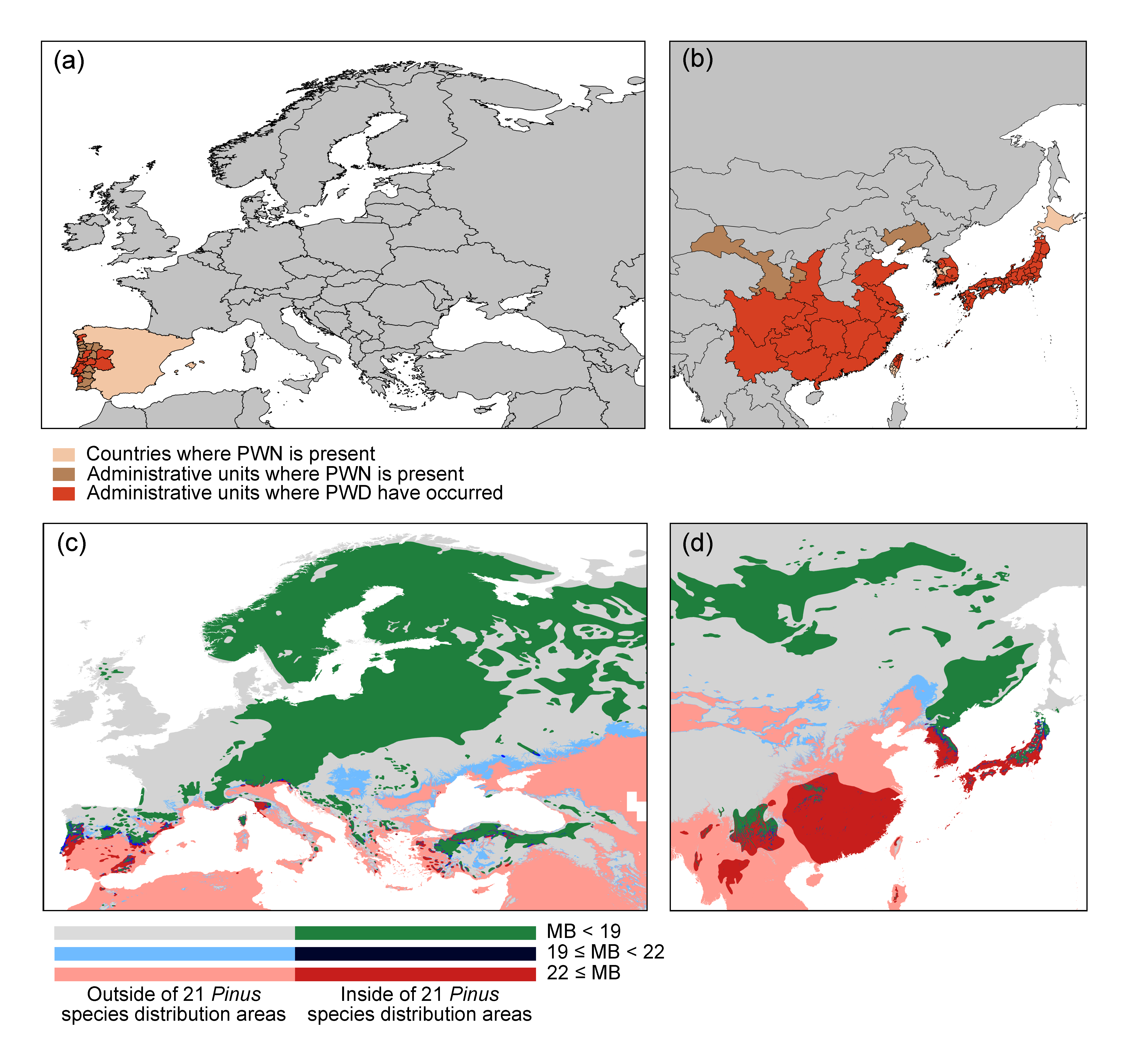

Supplement: S1 Fig — Distributions of PWN were determined according to EPPO [56], and occurrences of PWD in accordance to previous literature [29,30,34–38,57,58]. (TIF) [file pone.0182837.s001.tif]

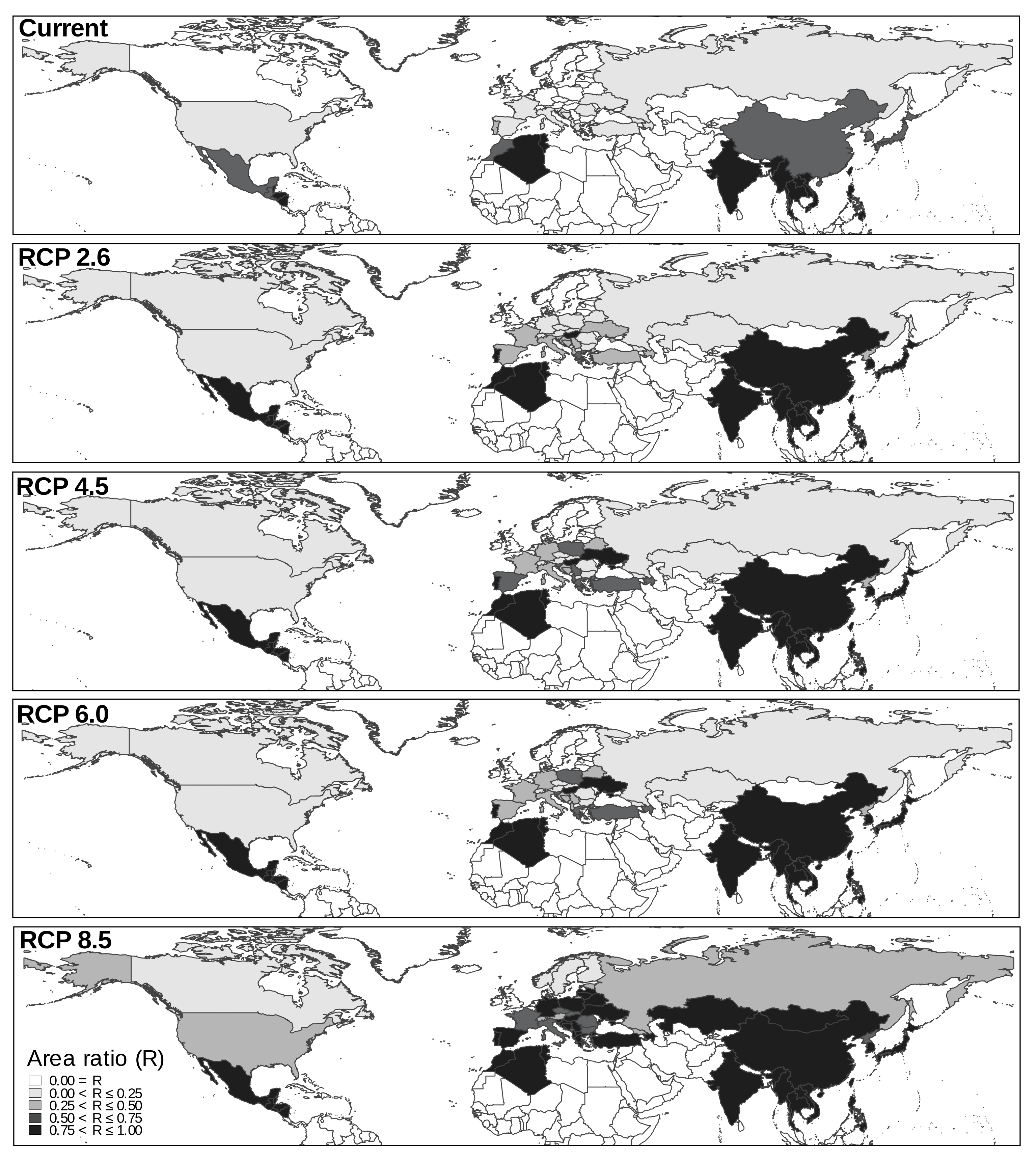

Supplement: S2 Fig — (TIF) [file pone.0182837.s002.tif]

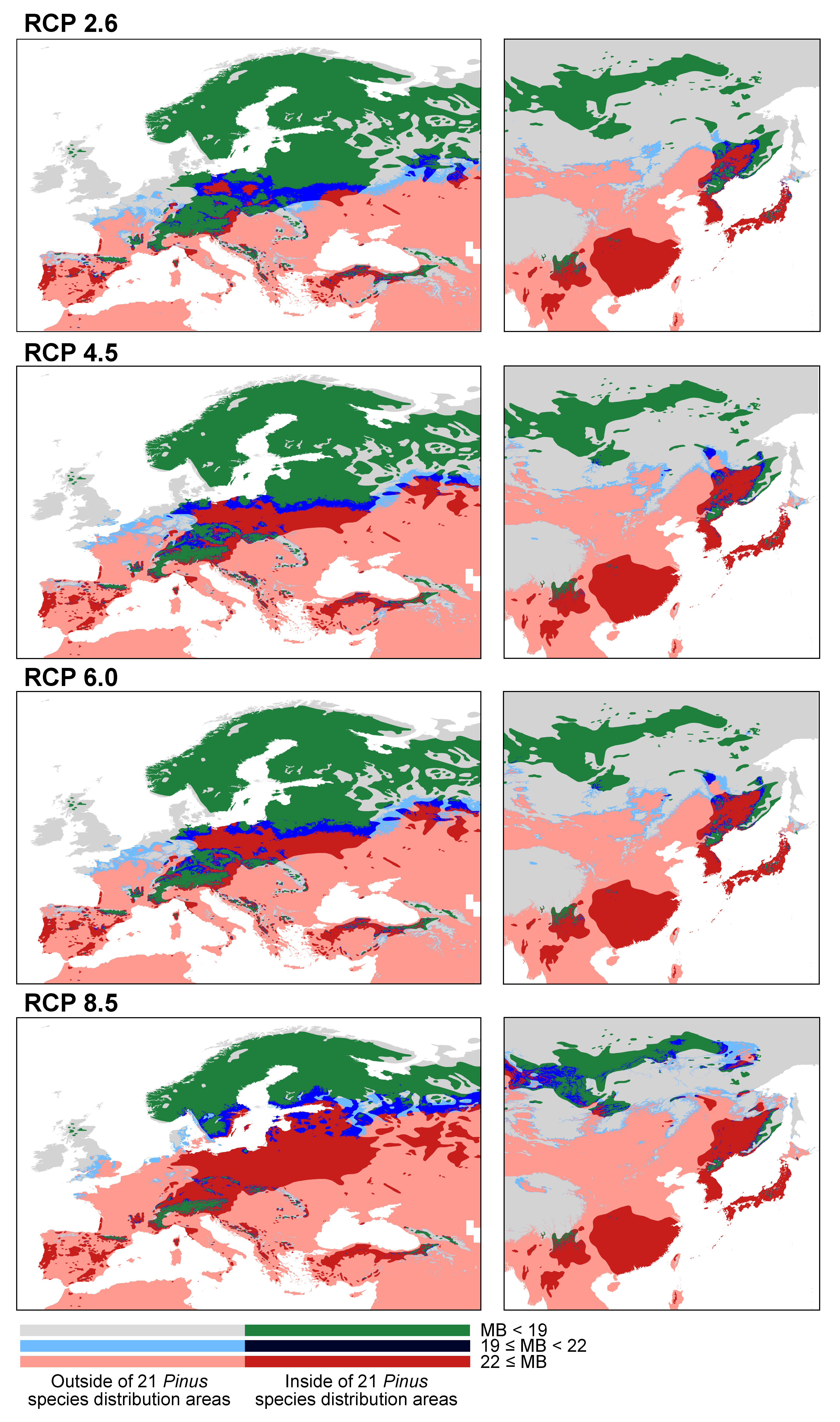

Supplement: S3 Fig — (TIF) [file pone.0182837.s003.tif]

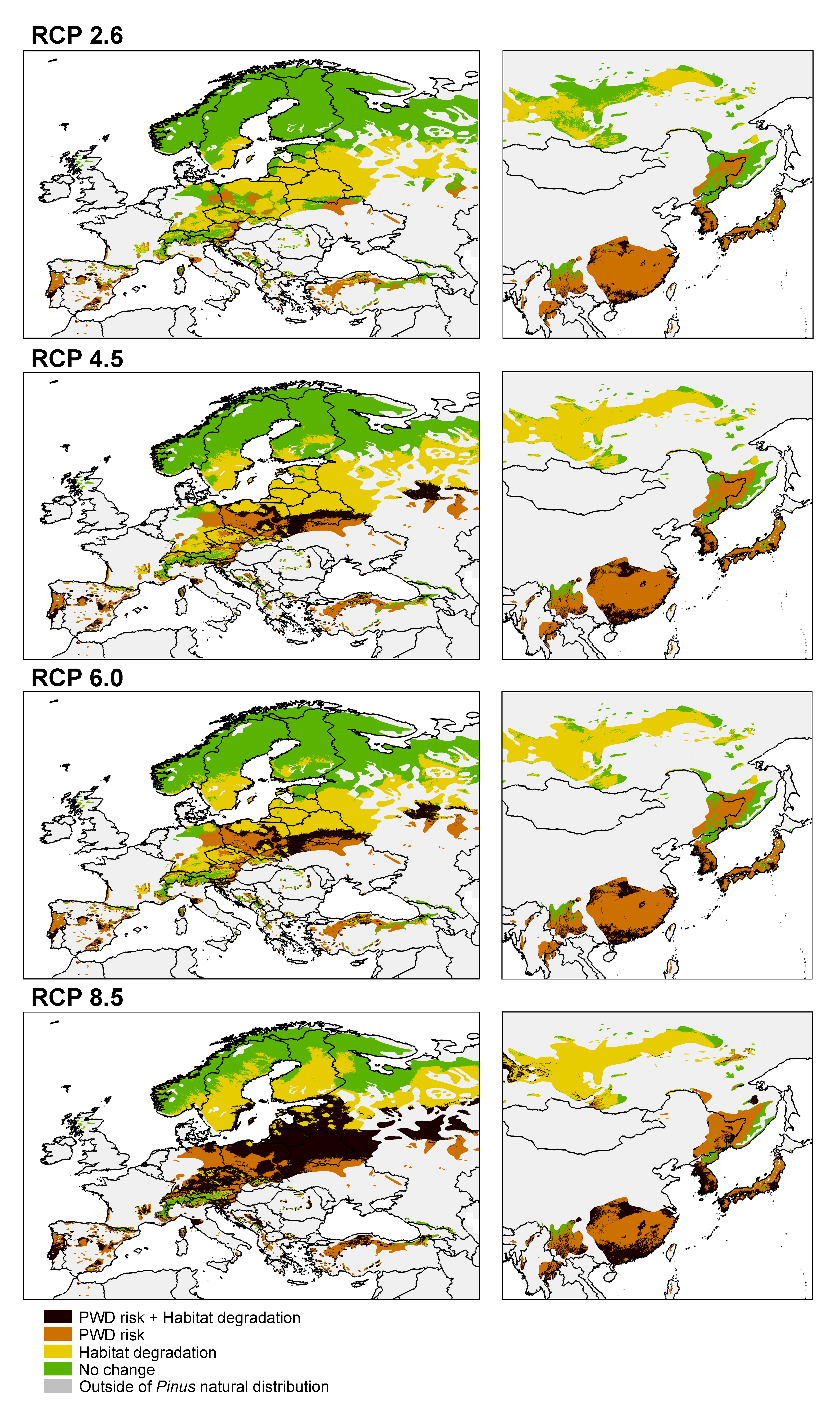

Supplement: S4 Fig — Areas integrating dark brown and yellow indicate regions vulnerable to habitat degradation. (TIF) [file pone.0182837.s004.tif]
